# Human parechovirus meningoencephalitis

**DOI:** 10.4102/sajr.v27i1.2589

**Published:** 2023-02-23

**Authors:** Pokhraj P. Suthar, Kathryn Hughes, Geetanjalee Kadam, Miral Jhaveri, Santhosh Gaddikeri

**Affiliations:** 1 Department of Diagnostic Radiology and Nuclear Medicine, Rush University Medical Center, Chicago, Illinois, United States of America

**Keywords:** meningoencephalits, human parechovirus, neonate, MRI, CSF

## Abstract

Human parechovirus-3 (HPeV-3) infection is one of the differential diagnoses of neonatal meningoencephalitis. A 13-day-old full-term female neonate presented with a seizure. Brain MRI showed classic imaging findings of the meningoencephalitis which was confirmed on cerebrospinal fluid analysis.

**Contribution:**

The HPeV-3 is an emerging pathogen for neonatal meningoencephalitis. The case in this study is unique with classic imaging findings, which are not routinely encountered in day-to-day practice. This case raises reader awareness.

## Introduction

The differential diagnosis of neonatal meningoencephalitis or sepsis is broad. Human parechovirus-3 (HPeV-3) is one of the differential diagnoses of neonatal meningoencephalitis or sepsis. It is an emerging pathogen that has been increasingly recognised as a causative agent in recent years.

The human parechoviruses are a family of viruses closely related to enteroviruses. Characteristically, young infants present with fever, irritability and on occasions, a diffuse rash. Severe disease can manifest as meningoencephalitis, seizures or sepsis-like presentations, including septic shock.

While MRI findings overlap in many of the bacterial and viral aetiologies, the presented case of human parechovirus infection in this report increases radiologists’ awareness of the characteristic MR brain imaging findings, providing a unique opportunity to be able to make a definitive diagnosis in clinically suspected cases of neonatal meningoencephalitis.

## Case report

A 13-day-old full-term female neonate presented with a seizure. At presentation, seizure semiology included rhythmic twitching of both upper and lower extremities with eye deviation. The neonate was feeding less but passing urine normally. On examination, the neonate was lethargic, with decreased tone in both upper extremities, weak grasp and head lag. The neonate had hypoglycaemia (neonatal heel prick glucose 24 mg/dL; normal reference range 46 mg/dL – 120 mg/dL) which was corrected with a glucose infusion. Continuous electroencephalogram (cEEG) monitoring revealed electrographic right centrotemporal seizures.

The baby was born at 37 weeks and 2 days to a 32-year-old G5P2 mother with APGAR scores of 8 and 9 at 1 min and 5 min, respectively. The antepartum and intrapartum course was unremarkable except for artificial rupture of the membranes for 2 h. Neonatal laboratory screening and blood culture were negative. Basic metabolic profile, magnesium and C-reactive protein were within normal limits. Serum procalcitonin was elevated. Lumbar puncture was performed, and cerebrospinal fluid (CSF) analysis demonstrated a decrease in CSF protein, normal glucose and no CSF pleocytosis. Ultrasound of the head was unremarkable (images not displayed).

**FIGURE 1:**
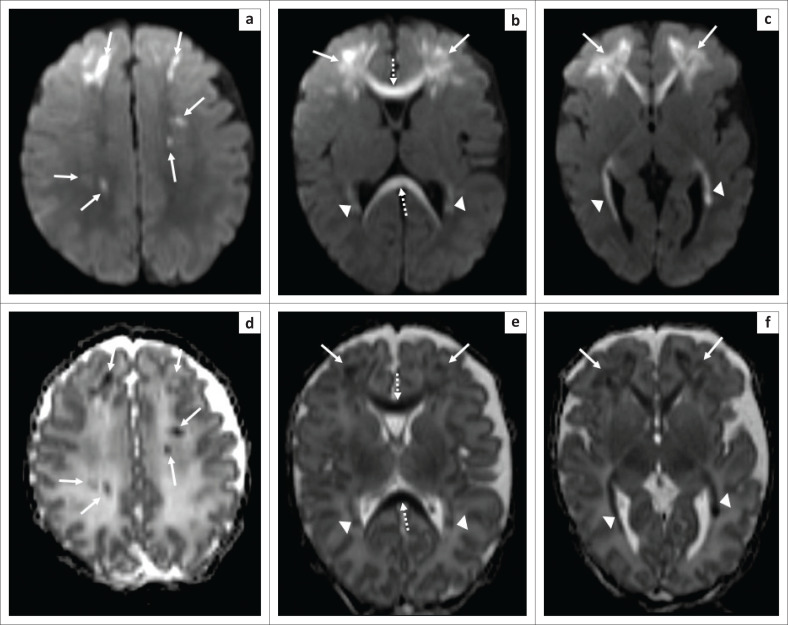
(a, b, c) Axial diffusion-weighted MRI of the brain (repetition time msec/echo time msec, 8500/100; flip angle, 90°; *b*-value = 1000 sec/mm^2^) and (d, e, f) corresponding axial apparent diffusion coefficient maps (8500/100; flip angle, 90°; *b-*value = 1000 sec/mm^2^) at the level of the high frontal lobes (a, d), basal ganglia (b, e) and peritrigonal white matter (c, f). Areas of restricted diffusion involving the bilateral supratentorial white matter, predominantly bilateral frontal lobes (solid white arrows on all images), corpus callosum (dashed white arrows on images [b]) and [e]) and bilateral peritrigonal periventiruclar white matter (solid white arrow heads on images [b], [c], [e] and [f]).

**FIGURE 2:**
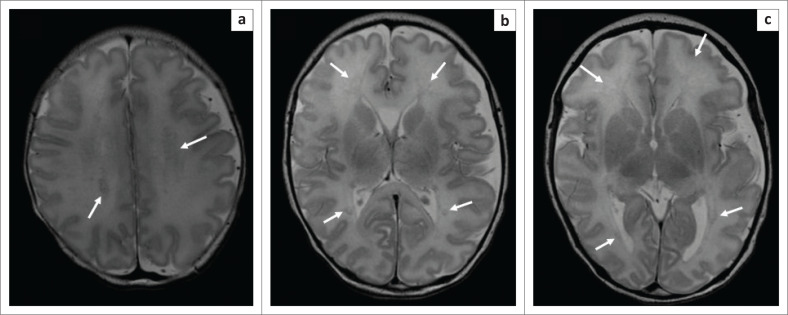
Axial T2-weighted MRI of the brain (repetition time msec/echo time msec, 3680/90, 3-mm section thickness) at the level of the high frontal lobes (a), basal ganglia (b) and peritrigonal white matter (c). The images show areas of T2 signal abnormality involving the corpus callosum and the bilateral supratentorial white matter, predominantly the bilateral frontal regions (solid white arrows on all images) with sparing of the thalami.

**FIGURE 3:**
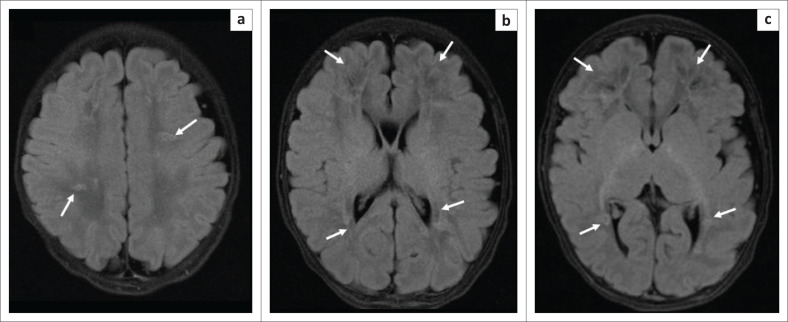
Axial fluid-attenuated inversion recovery MRI of the brain (inversion time 2500, repetition time msec/echo time msec, 9000/112, 3-mm section thickness) at the level of the high frontal lobes (a), basal ganglia (b) and peritrigonal white matter (c). Images demonstrate areas of FLAIR signal abnormality involving the corpus callosum and the bilateral supratentorial white matter, predominantly the bilateral frontal regions (solid white arrows on all images) with sparing of the thalami.

**FIGURE 4:**
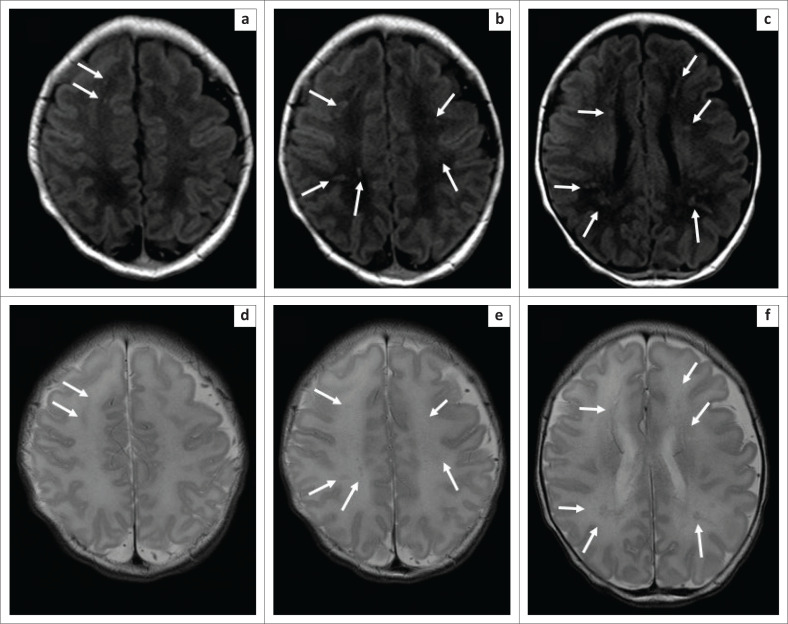
Axial unenhanced T1-weighted MRI of the brain (repetition time msec/echo time msec, 300/2.52, 3-mm section thickness) at the level of centrum semiovale and corona radiata (a, b, c) with corresponding axial T2-weighted MRI of the brain (repetition time msec/echo time msec, 3680/90, 3-mm section thickness) (d, e, f). There were areas of T1 and T2 shortening following the distribution of deep medullary veins (solid white arrows on all images), more evident on the T1-weighted images.

**FIGURE 5:**
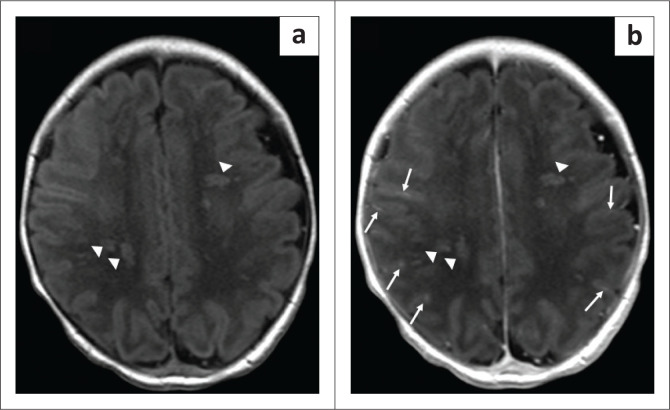
Axial T1-weighted MRI of the brain at the level of centrum semiovale (repetition time msec/echo time msec, 300/2.52, 3-mm section thickness), unenhanced (a) and contrast-enhanced (b) after intravenous injection of 1 mL of gadoteridol (ProHance; Bracco). There were areas of non-enhancing signal abnormality in the bilateral supratentorial frontal white matter (solid white arrow heads on images [a] and [b]), corresponding to the restricted diffusion. Subtle leptomeningeal contrast enhancement seen (solid white arrows on image b).

Multiplanar multi-sequence MRI of the brain was performed for further evaluation, revealing areas of restricted diffusion and T2 fluid-attenuated inversion recovery (FLAIR) signal abnormality involving the entire corpus callosum and bilateral supratentorial white matter, predominantly in the frontal regions with sparing of the thalami (Figure 1, Figure 2 and Figure 3). There were areas of T1 and T2 shortening in the distribution of the deep medullary veins (Figure 4). T1-weighted contrast-enhanced MRI indicated faint minimal leptomeningeal contrast enhancement without parenchymal contrast enhancement (Figure 5). Based on the imaging findings, the diagnosis of an infectious process such as meningoencephalits was considered. The CSF meningitis encephalitis panel was positive for human parechovirus.

## Discussion

Closely related to enteroviruses, human parechoviruses comprise a genus within the Picornaviridae family.^1,2,3^ Up to 19 different parechovirus genotypes have been identified with varying clinical presentations.^3,4^ Transmission is predominantly via the oral-faecal route.^1^ Although other genotypes are often associated with mild symptoms, HPeV-3 is an emerging pathogen that has been increasingly recognized as a causative agent of neonatal meningoencephalitis and sepsis.^1,2^ The pathophysiology of the HPeV-3 is not clearly understood.^4^

Human parechovirus-3 most commonly infects children less than 3 months of age, with infections uncommon among patients older than 10 years.^1,2^ Clinically, patients with HPeV-3 infection may present with mild gastrointestinal or respiratory symptoms, rash or severe disease such as sepsis and central nervous system (CNS) infection.^1,2,5^ Risk factors for severe disease may include prematurity and age less than 28 days.^2^ In this study, a 13-day old patient was at risk of severe HPeV-3 disease and presented with signs of CNS infection. Treatment of HPeV-3 is supportive, although patients often empirically receive antibiotics and acyclovir because of overlapping presentations with bacterial and herpes simplex virus (HSV) infection.^5^

Human parechovirus-3 infection may be confirmed via reverse transcriptase real-time polymerase chain reaction (RT-PCR) using stool, blood, respiratory samples or CSF, while characteristic imaging plays a supportive role in diagnosis.^1,3^ Cranial ultrasound findings are non-specific and can be insensitive early in the disease course.^2^ Parechovirus meningoencephalitis is best evaluated with MRI.^2^ Characteristic imaging findings include bilateral supratentorial white matter abnormalities with corresponding restricted diffusion.^4,6,7^ Involvement along the deep medullary veins, thalamus and entire corpus callosum with sparing of the basal ganglia and posterior fossa have also been reported.^4^ The preference for supratentorial white matter suggests supratentorial white matter neuroaxonal trophism. Signal abnormality along the course of the deep medullary vein suggests perivenular invasion or venous ischemia. The presented case demonstrates these classic imaging findings of T2-FLAIR hyperintensity and corresponding restricted diffusion involving the subcortical white matter and entire corpus callosum, with some lesions along the deep medullary veins. The basal ganglia and posterior fossa were spared. Head ultrasound, in this study, was normal.

Differential diagnoses include hypoxic-ischemic encephalopathy (HIE), molybdenum cofactor deficiency (MCD) and isolated sulfite oxide deficiency (ISOD). A major cause of neonatal morbidity and mortality, HIE typically involves one of two main imaging patterns on MRI, which are strong predictors of neurodevelopmental outcome: cortical watershed and deep grey matter.^8,9^ In contrast to parechovirus encephalitis, which predominantly involves the subcortical white matter, the watershed pattern of HIE involves the cortex along the borders of the major arterial vascular territories.^9^ In addition, the deep grey matter pattern of HIE affects the basal ganglia and thalamus, whereas HPeV-3 encephalitis often spares the basal ganglia.^4,9^

Molybdenum cofactor deficiency is a rare autosomal recessive disease, which can mimic HIE on MRI.^10^ It results from decreased production of molybdenum, a cofactor for three important enzymes: sulfite oxidase, xanthine dehydrogenase and aldehyde oxidase.^11^ The resultant toxicity from elevated neuronal levels of sulfite in MCD causes progressive, fatal neurological decline, with a median survival of 36 months.^11^ Similar to HIE, the MRI findings of MCD include diffuse cerebral oedema with involvement of the deep grey matter, followed by cerebral and cerebellar atrophy and ventriculomegaly.^11^ In contrast, the characteristic HPeV-3 imaging findings involving the supratentorial white matter with sparing of the basal ganglia and posterior fossa, can thus help to differentiate MCD from HPeV-3 encephalitis.

Like MCD, ISOD is a rare, fatal autosomal recessive disorder that often presents with seizures and progressive neurological decline.^11^ Sulfite oxidase utilizes molybdenum as a cofactor, and thus MCD and ISOD may have similar clinical and imaging features, with laboratory confirmation distinguishing between the two conditions.^11^ On MRI, ISOD may indicate signal abnormalities involving the cortex, subcortical white matter, thalami and basal ganglia, with marked cerebral volume loss and cystic encephalomalacia over time.^11^ The condition can thus be differentiated from HPeV-3 by the striking cystic changes and involvement of the basal ganglia with ISOD, compared with the supratentorial white matter predominance and sparing of the basal ganglia seen with HPeV-3.

## Conclusion

Severe infection with HPeV-3 can manifest as meningoencephalitis, seizures or sepsis-like presentations, including septic shock. While MR brain imaging findings overlap in bacterial and viral aetiologies of meningitis, human parechovirus infection displays characteristic findings which can support a definitive diagnosis in clinically suspected cases. As only a few cases are documented in the literature and cases are infrequently encountered in day-to-day practice, radiologists should familiarize themselves with the MR imaging findings in this condition.
